# Co-Improvement in Electrocatalytic Hydrogen Evolution Performance of MoS_2_ by Ni Doping and Graphene Oxide Compounding

**DOI:** 10.3390/molecules30040963

**Published:** 2025-02-19

**Authors:** Guiquan Guo, Yuqin Li, Shujiao Zhang, Cuijuan Xing, Qi Wang

**Affiliations:** School of Chemical Engineering and Biotechnology, Xingtai University, Xingtai 054001, China; 202320018@xttc.edu.cn (Y.L.); 201420358@xttc.edu.cn (S.Z.); 201420357@xttc.edu.cn (C.X.); 202420008@xttc.edu.cn (Q.W.)

**Keywords:** molybdenum disulfide (MoS_2_), Ni doping, graphene oxide (GO), electrocatalysis, hydrogen evolution reaction

## Abstract

Molybdenum disulfide (MoS_2_) is a promising catalyst for hydrogen evolution through water electrolysis with low cost and high efficiency, but its hydrogen evolution performance can be further improved. Using sodium molybdate (Na_2_MoO_4_·2H_2_O) and thiourea (NH_2_CSNH_2_) as raw materials, MoS_2_ was prepared by the hydrothermal method. Ni-doped MoS_2_(Ni-MoS_2_) was prepared by using nickel dichloride dihydrate (NiCl_2_·2H_2_O) as a Ni source and doping Ni into MoS_2_ by the hydrothermal method. Under the conditions of different temperatures (190 °C, 200 °C, and 210 °C) and different Ni doping molar ratios (2%, 3%, and 4%), the optimum temperature and doping ratio of the prepared materials were explored by conducting a hydrogen evolution reaction (HER) by the electrolysis of water. The results showed that the optimum preparation temperature was 200 °C and the optimum molar ratio of Ni doping was 3%. Graphene oxide (GO) was obtained by oxidation of graphite (G), and then Ni-MoS_2_/GO was prepared by the hydrothermal method with Ni-MoS_2_ and GO. The performance of HER was tested. The materials were characterized by X-ray diffraction (XRD), scanning electron microscope (SEM), high-resolution transmission electron microscope (HRTEM), and X-ray photoelectron spectroscopy (XPS). The results show that the composite Ni-MoS_2_/GO has good HER performance, which is better than that of MoS_2_ or Ni-MoS_2_. In 0.5 M H_2_SO_4_ solution, the η_10_ is as low as 196 mV, the Tafel slope is 122 mV/dec, the C_dl_ is 13.98 mF/cm^2^, and it has good stability. The enhancement of electrocatalytic activity is mainly due to the doping of a small amount of Ni, which increases the defects of the catalyst and forms more active sites. GO improves the conductivity of the material. Ni doping and GO compounding promote the HER performance of MoS_2_.

## 1. Introduction

Energy shortages and environmental pollution are significant problems that puzzle human beings; however, the development of green and environmentally friendly hydrogen energy can address these issues. Electrocatalytic hydrogen evolution (HER) is the most promising method to obtain hydrogen energy [1,2,3,4]. Platinum, a noble metal, is a catalyst with good hydrogen evolution performance, and its superior hydrogen production ability is attributed to its best hydrogen adsorption free energy in the HER reaction [5,6], but its insufficient reserves and expensive price limit its industrial application in the hydrogen evolution reaction by the electrolysis of water [7]. Therefore, the development of cheap, efficient, and industrialized electrocatalysts for hydrogen evolution is an urgent need that can be addressed by hydrogen energy [8,9].

Nano-structured MoS_2_ has a low price and good electrocatalytic hydrogen evolution performance. Its catalytic activity mainly comes from the edge of MoS_2_, and the adsorption free energy of hydrogen at the edge of MoS_2_ is close to that of Pt and other catalysts [10]. However, the low conductivity of MoS_2_ affects the electron transfer in the HER process. In addition, MoS_2_ easily accumulates between layers, which leads to a decrease in its catalytic active sites [11]. Therefore, improving the conductivity of nano-structured MoS_2_, increasing the number of catalytic active sites [12], and improving the inherent catalytic activity of active sites are important ways to improve its electrocatalytic hydrogen evolution performance.

Graphene has high conductivity, a large specific surface area, and good flexibility [13], and it can be used as an ideal substrate for growing MoS_2_ nano-layers. Li et al. [14,15] prepared MoS_2_/graphene composites by a solvothermal reaction and found that graphene not only improves the conductivity of composite catalysts but also inhibits the agglomeration between MoS_2_ layers, producing MoS_2_/graphene composites with high HER electrocatalytic activity. Graphene oxide (GO) has a structure and performance similar to that of graphene. Graphene oxide (GO) has a large specific surface area and contains various oxygen-containing functional groups on its surface, so it shows super solubility in both the water phase and in organic solvents [16,17]. In the field of electrochemistry, a nano-heterojunction structure prepared by chemically loading WO_3_ and GO showed excellent electrocatalytic activity for electrolytic hydrogen production in an acidic environment [18].

In addition, doping is considered to be one of the most effective ways to regulate the electrochemical hydrogen evolution performance of a catalyst. Appropriate heteroatom doping can optimize the electronic structure of MoS_2_, activate its in-plane hydrogen evolution activity, increase the number of active sites for hydrogen evolution, and thus reduce its hydrogen evolution reaction barrier. At the same time, heteroatomic doping can also regulate the energy band structure of MoS_2_ and improve its conductivity, thus increasing its hydrogen evolution reaction rate [19,20,21,22,23,24,25]. To date, many metal atoms (such as Fe [20], Co [21], Ni [22], V [23], Mn [24], or Zn [25]) have been proven to be effective dopants in improving the electrocatalytic hydrogen evolution performance of MoS_2_. Bonde et al. [26,27] found through theoretical calculation that the free energy of hydrogen adsorption (ΔG_H_ = 0.08 eV) at the edge of Mo is lower than that at the edge of S (ΔG_H_ = 0.18 eV) and the hydrogen evolution reaction mainly occurs at the Mo edge of MoS_2_, but after Co doping, the ΔG_H_ at the edge of S decreases to 0.10 eV, and the intrinsic activity of the catalytic active site of MoS_2_ is improved. The research of Merki et al. [28,29] shows that doping transition metal elements such as Ni, Fe, and Cu can also reduce the free energy of hydrogen adsorption at the edge of MoS_2_ and improve its electrocatalytic activity.

In order to further improve the electrocatalytic hydrogen evolution performance of MoS_2_ in view of the doping of metal elements such as Ni and the excellent performance of RGO or GO in the electrochemical field, relevant studies have been reported recently. As reported in [30,31,32], the electrocatalytic hydrogen evolution performance of MoS_2_ was improved by Ni doping and RGO compounding, with η_10_ as low as 168 mV [32], the initial potential as low as 85 mV [31], and the Tafel slope as low as 71.3 mV·dec^−1^ [31]. As reported in [33], the electrocatalytic hydrogen evolution performance of MoS_2_ was also improved by Ni doping and GO compounding, with η_10_ as low as 33 mV. However, the studies in [32,33] are based on an alkaline system, and the η_10_ reported in [30] is high, reaching 398 mV. Moreover, the effects of temperature and doping ratio on the properties of the prepared materials have not been thoroughly and systematically discussed. Therefore, in this study, MoS_2_, Ni-MoS_2_, and Ni-MoS_2_/GO composites were prepared by the hydrothermal method. By means of XRD, SEM, TEM, and XPS and electrochemical performance testing techniques, such as LSV, CV, Tafel slope, C_dl_, and EIS, the effects of different preparation conditions, such as reaction temperature, Ni doping amount, and graphene oxide participation, on the structure of MoS_2_ and its electrocatalytic hydrogen evolution performance in an acidic environment were investigated. Good research results are obtained, which is expected to provide reference for the research in the field of electrocatalytic hydrogen evolution.

## 2. Results and Discussion

### 2.1. Effect of Preparation Temperature on Structure and HER Properties of Ni-MoS_2_

#### 2.1.1. Material Structure and Morphology

**Figure 1:**
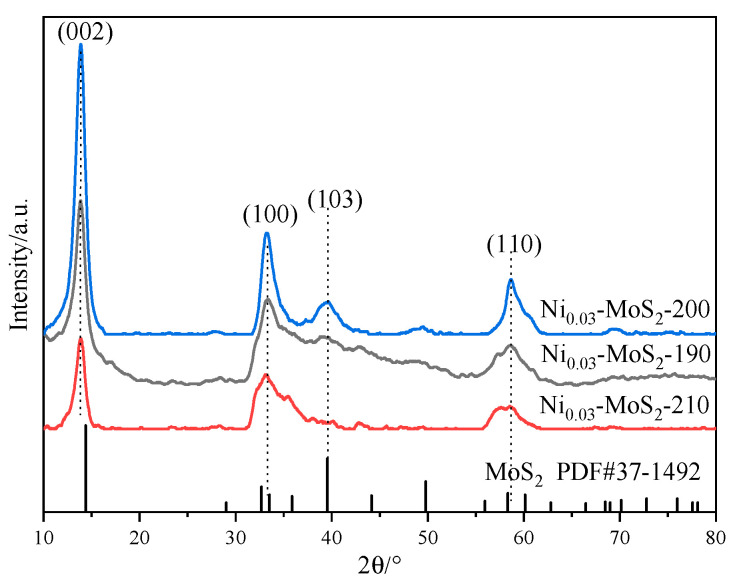
XRD patterns of Ni-MoS_2_ prepared at different temperatures.

Figure 1 reveals the X-ray diffraction (XRD) patterns of three materials, Ni_0.03_-MoS_2_-190, Ni_0.03_-MoS_2_-200, and Ni_0.03_-MoS_2_-210, which were prepared by MoS_2_ doped with 3%Ni at 190 °C, 200 °C, and 210 °C. The outlines of these three curves are generally similar, but there are also slight differences. The diffraction peaks located at approximately 13.9°, 33.7°, 39.4°, and 59.1° correspond to the (002), (100), (103), and (110) crystal planes of 2H-MoS_2_ (JCPDS No. 37-1492), respectively. However, compared with pure 2H-MoS_2_, the peaks of the (002) crystal face all moved by 0.5° in the direction of small angle, indicating that Ni entered the crystal lattice of MoS_2_, which made the spacing between (002) crystal faces larger [34,35]. The intensity of each peak first increases and then decreases with the increase in temperature, which shows that the crystallinity of the material will change with the temperature, especially the intensities of the peaks of (103) and (110) crystal planes, which are quite different and which increase from 190 °C to 200 °C, but when the temperature continues to increase to 210 °C, the peak of the (103) crystal plane disappears and the peak of the (110) crystal plane is weakened. The exposure degree of the (103) and (110) crystal planes and the crystallization degree of the material may have an influence on the performance of the material. In addition, the diffraction peak of Ni is not clearly displayed, which is related to its low content.

**Figure 2:**
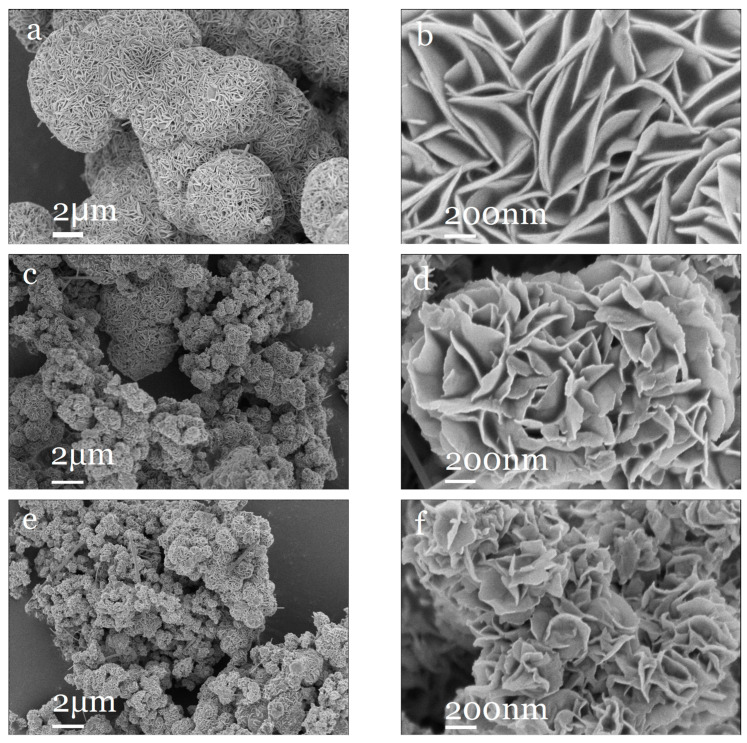

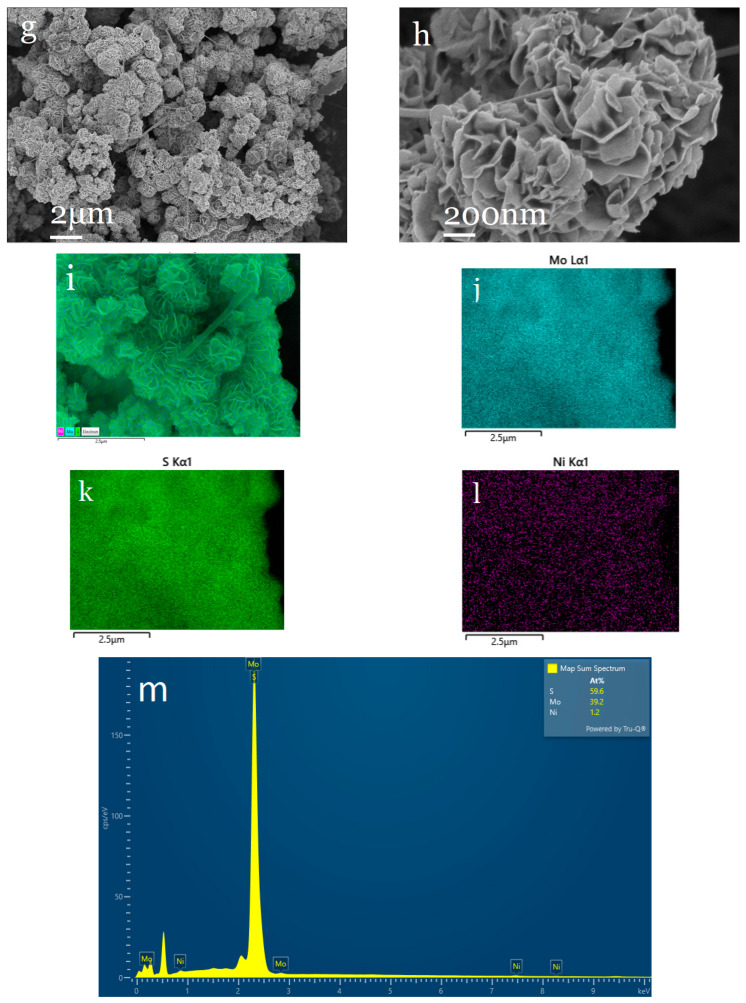
SEM images of (**a**,**b**) MoS_2_, (**c**,**d**) Ni_0.03_-MoS_2_-190, (**e**,**f**) Ni_0.03_-MoS_2_-200, (**g**,**h**) Ni_0.03_-MoS_2_-210, and (**i**–**m**) EDS images of Ni_0.03_-MoS_2_-200.

Figure 2 shows the scanning electron microscope images of four materials: MoS_2_, Ni_0.03_-MoS_2_-190, Ni_0.03_-MoS_2_-200, and Ni_0.03_-MoS_2_-210. It can be observed from Figure 2a,b that MoS_2_ is a regular nanosheet, and micron flower balls with uniform size are formed by self-assembly. Moreover, the overall morphology of Ni-doped MoS_2_ is similar to MoS_2_ (Figure 2c–h), but the regularity of nanosheets is weakened and the size of micron flower balls is reduced. Compared with Ni_0.03_-MoS_2_-190 prepared at 190 °C, Ni_0.03_-MoS_2_-200 prepared at 200 °C has irregular nano-petals, more defects, and a smaller micron flower ball size, which may be due to the fact that the doping of Ni atoms into the MoS_2_ lattice inhibits the growth of MoS_2_ grains [36]. The decrease in the flower ball size means an increase in its specific surface area, indicating that more active sites are exposed, which is beneficial to the improvement of hydrogen evolution performance. Compared with Ni_0.03_-MoS_2_-200 prepared at 200 °C, Ni_0.03_-MoS_2_-210 prepared at 210 °C has almost the same nano-petal regularity and micron flower ball size, so it is difficult to distinguish the difference with naked eyes. Obviously, the microstructure and size of Ni-MoS_2_ can be significantly changed by adjusting the temperature. In addition, the sample Ni_0.03_-MoS_2_-200 was analyzed by energy spectrum surface scanning, and the results are shown in Figure 2i–m. It can be clearly seen that Mo, S, and Ni are evenly distributed in space, which further proves that Ni has been successfully doped into the MoS_2_ lattice.

#### 2.1.2. Electrocatalytic Hydrogen Evolution Performance

**Figure 3:**
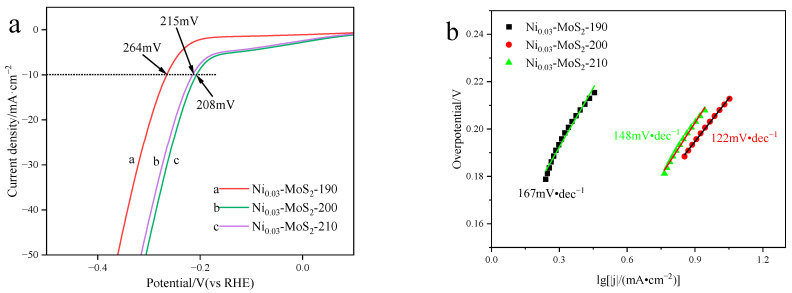
(**a**) LSV diagram (the black dotted line represents a current density of 10 mA·cm^−2^) and (**b**) Tafel slope diagram of Ni_0.03_-MoS_2_-190, Ni_0.03_-MoS_2_-200, and Ni_0.03_-MoS_2_-210 prepared at 190 °C, 200 °C, and 210 °C, respectively.

Figure 3a shows the LSV curves of Ni_0.03_-MoS_2_-190, Ni_0.03_-MoS_2_-200, and Ni_0.03_-MoS_2_-210 and can intuitively compare their differences in electrochemical performance. At a current density of 10 mA/cm^2^, Ni_0.03_-MoS_2_-200 exhibits an overpotential of 208 mV, which is the lowest value among the three samples. It shows that the electrocatalytic properties of the prepared materials are different at different temperatures, and the Ni_0.03_-MoS_2_-200 material prepared at 200 °C has the best performance. Moreover, when the overpotential is 300 mV, the current density of Ni_0.03_-MoS_2_-200 is the largest, which is related to the positive correlation between the cathode current density and hydrogen production. Figure 3b provides a comparison of the Tafel slopes of three materials. Generally speaking, the lower the slope of the Tafel curve (η = blg∣j∣ + a, where η is overpotential, j is current density, and b is slope), the better the reaction kinetics of the catalyst [37]. The results show that the Tafel slopes of Ni_0.03_-MoS_2_-190, Ni_0.03_-MoS_2_-200, and Ni_0.03_-MoS_2_-210 are 167 mV/dec, 122 mV/dec, and 148 mV/dec, respectively. Usually, when the slope of Tafel is about 120 mV/·dec^−1^, the corresponding reaction mechanism is the Volmer–Heyrovsky step. The Tafel slopes of these three materials are all around 120 mV/·dec^−1^, which shows that their reaction mechanisms are the same. In contrast, the Tafel slope of Ni_0.03_-MoS_2_-200 is obviously lower, which demonstrates that its Volmer–Heyrovsky dynamic process is faster.

**Figure 4:**
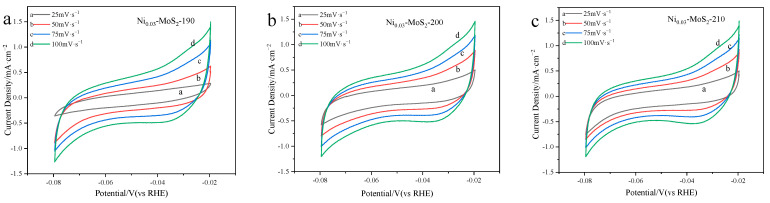
Cyclic voltammetry of (**a**) Ni_0.03_-MoS_2_-190, (**b**) Ni_0.03_-MoS_2_-200, and (**c**) Ni_0.03_-MoS_2_-210 prepared at 190 °C, 200 °C, and 210 °C, respectively.

**Figure 5:**
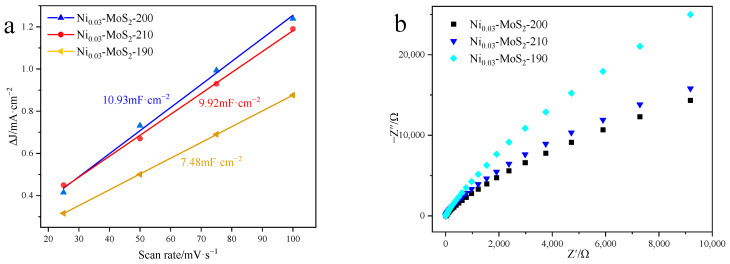
(**a**) C_dl_ diagram and (**b**) electrochemical impedance diagram of Ni_0.03_-MoS_2_ prepared at different temperatures.

Figure 4 shows the cyclic voltammetry of three catalytic materials: Ni_0.03_-MoS_2_-190, Ni_0.03_-MoS_2_-200, and Ni_0.03_-MoS_2_-210. It can be noted that with the increase in scanning speed, the current density of the three materials shows a regular increase. Usually, the larger the area enclosed by the CV curve, the more substances are reacting; that is, the larger the amount of charge generated by the reaction, which implies that the electrochemical surface area (ECSA) of the electrode is larger. As can be seen from Figure 4, when the scanning speed is 25 mV·s^−1^, the enclosed area of the Ni_0.03_-MoS_2_-190 curve is the smallest, indicating that its ECSA is the smallest. The results of Ni_0.03_-MoS_2_-200 and Ni_0.03_-MoS_2_-210 are very close, which cannot be distinguished by naked eyes. The difference in their ECSA (i.e., capacitance) are shown in Figure 5a.

Figure 5a clearly shows the differences in the capacitance characteristics of three materials, namely Ni_0.03_-MoS_2_-190, Ni_0.03_-MoS_2_-200, and Ni_0.03_-MoS_2_-210. Combined with the data in Figure 4, we find that the capacitance values of these three materials are 7.48 mF/cm^2^, 10.93 mF/cm^2^, and 9.92 mF/cm^2^ by fitting the slopes. It is particularly noteworthy that the capacitance value of Ni_0.03_-MoS_2_-200 is as high as 10.93 mF/cm^2^, indicating that it has a larger effective active area, further revealing that MoS_2_ prepared at different reaction temperatures contains different defects and exposes different active sites.

Figure 5b shows the electrochemical impedance properties of three materials Ni_0.03_-MoS_2_-190, Ni_0.03_-MoS_2_-200, and Ni_0.03_-MoS_2_-210. In theory, a smaller diameter of the high-frequency semicircle indicates a lower charge transfer resistance, thereby resulting in a faster rate of the electrocatalytic reaction. From the observation diagram, it can be found that Ni_0.03_-MoS_2_-200 has the smallest semicircle diameter, which indicates that its resistance is the lowest in the process of charge transfer, which is conducive to the effective transmission of electrons and the performance of the best catalytic activity.

Comparing Ni_0.03_-MoS_2_-190, Ni_0.03_-MoS_2_-200, and Ni_0.03_-MoS_2_-210 comprehensively, Ni_0.03_-MoS_2_-200 undoubtedly shows the best electrical properties, which may be related to its exposed (103) crystal plane, irregular nano petals, and smaller micron flower balls.

### 2.2. Effect of Ni Doping Amount on Structure and HER Properties of Ni-MoS_2_

#### 2.2.1. Material Structure and Morphology

**Figure 6:**
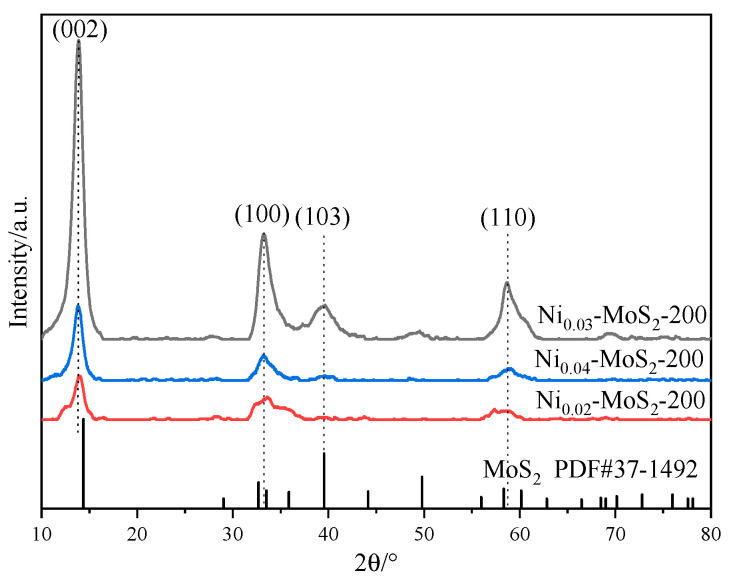
XRD patterns of Ni-MoS_2_-200 prepared by different Ni doping ratios.

Figure 6 shows the XRD diffraction patterns of Ni_0.02_-MoS_2_-200, Ni_0.03_-MoS_2_-200, and Ni_0.04_-MoS_2_-200 samples prepared with the molar ratios of Ni to Mo in the raw materials of 0.02: 1, 0.03: 1, and 0.04: 1, respectively. The outlines of these three curves are generally similar, but there are also slight differences. The diffraction peaks located at approximately 13.9°, 33.7°, 39.4°, and 59.1° correspond to the (002), (100), (103), and (110) crystal planes of 2H-MoS_2_ (JCPDS No. 37-1492), respectively. However, compared with pure 2H-MoS_2_, the peaks of the (002) crystal face all moved by 0.5° to a small angle direction, indicating that Ni entered the crystal lattice of MoS_2_, which made the spacing between the (002) crystal faces larger. With the increase in the Ni doping ratio, the intensity of each peak first increases and then decreases, indicating that the crystallinity of the material will change with the doping ratio, especially the peak intensities of the (103) and (110) crystal planes are quite different. When the doping ratio increases from 0.02: 1 to 0.03: 1, the peak intensities increase, However, when the doping ratio continually increases to 0.04: 1, the peak intensities of the (103) and (110) crystal planes decrease again. The exposure degree of the (103) and (110) crystal planes and the crystallization degree of the material may have an influence on the material performance. In addition, the diffraction peak of Ni is not clearly displayed in XRD, which is related to its low content.

**Figure 7:**
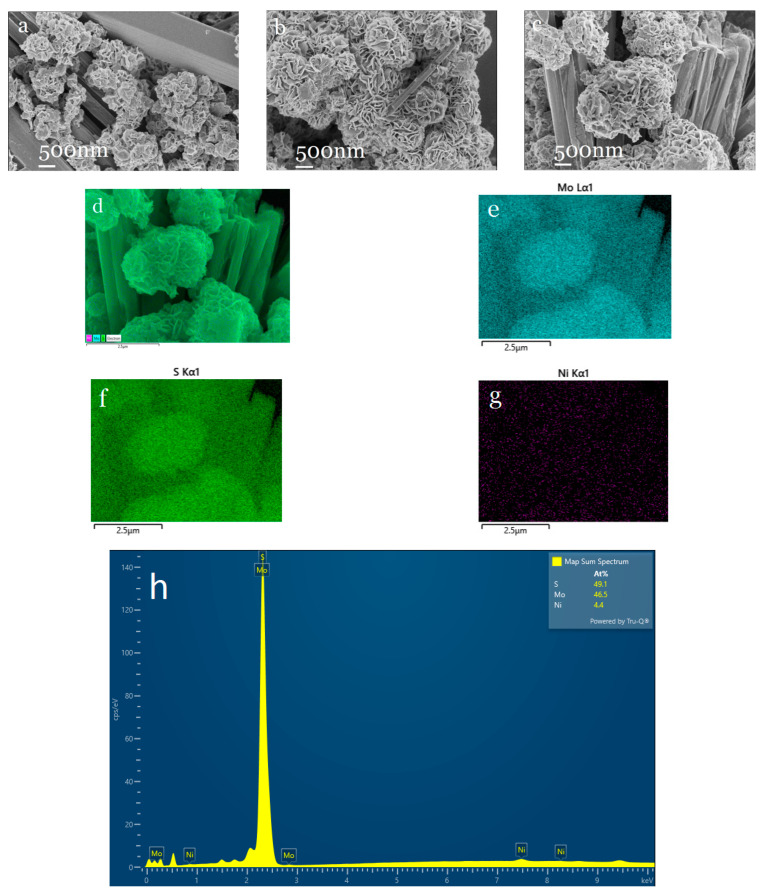
SEM images of (**a**) Ni_0.02_-MoS_2_-200, (**b**) Ni_0.03_-MoS_2_-200, (**c**) Ni_0.04_-MoS_2_-200, and (**d**–**h**) EDS images of Ni_0.04_-MoS_2_-200.

Figure 7a–c show the microscopic images of Ni-MoS_2_-200 samples with different doping ratios observed by scanning electron microscopy. Ni_0.03_-MoS_2_-200 prepared with a 3% doping ratio mainly presents micron flower balls assembled with nano-petals, which are uniform in size and have very few rod-like structures. However, when the doping ratio of Ni in the sample is 2% or 4%, many rod-like structures appear, and the surface of rod-like structures is smooth and flat, obviously resulting in a smaller specific surface area than that of micron flower balls assembled with nano-petals. The results strongly prove that the doping ratio of Ni has a significant influence on the microstructure and morphology of Ni-MoS_2_-200. Only when the doping ratio is appropriate can a uniform micron flower ball structure assembled by nano petals be presented. In addition, we scanned the Ni_0.04_-MoS_2_-200 sample using an energy spectrum. As shown in Figure 7d–h, we can clearly see the existence of Mo, S, and Ni, meaning Ni was successfully doped into MoS_2_, and the compositions of the rod-like structure and flower-ball structure are the same, but the morphologies are different.

#### 2.2.2. Electrocatalytic Hydrogen Evolution Performance

**Figure 8:**
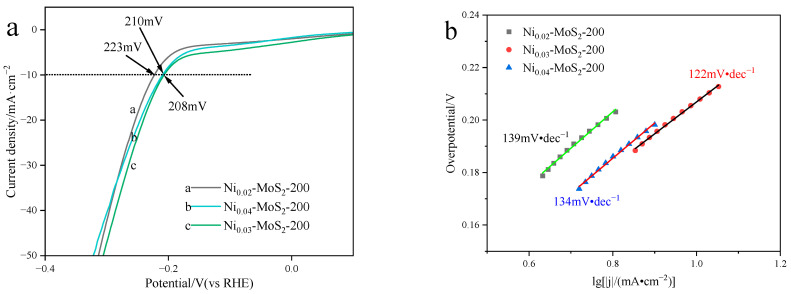
(**a**) LSV diagram (the black dotted line represents a current density of 10 mA·cm^−2^) and (**b**) Tafel slope diagram of Ni_0.02_-MoS_2_-200, Ni_0.03_-MoS_2_-200, and Ni_0.04_-MoS_2_-200 prepared with Ni doping ratios of 0.02, 0.03, and 0.04, respectively.

Figure 8a shows the linear scanning voltammogram (LSV) of the Ni-MoS_2_-200 material when the doping ratio of Ni is 2%, 3%, and 4%. When the cathode current density reaches 10 mA/cm^2^, the overpotential is 223 mV, 208 mV, and 210 mV, respectively, and the overpotential of Ni_0.03_-MoS_2_-200 with a 3% Ni doping ratio is the lowest, indicating that the doping ratio has an influence on the electrical properties of the material. Based on the data in Figure 8a, a Tafel slope diagram (Figure 8b) was made. It reveals the Tafel slope differences in Ni-MoS_2_-200 with doping ratios of 2%, 3%, and 4%, which are 139 mV/dec, 122 mV/dec, and 134 mV/dec, respectively. Tafel slopes of three materials are all around 120 mV/·dec^−1^, which shows that their reaction mechanisms are the same. In contrast, the Tafel slope of Ni_0.03_-MoS_2_-200 is lower, which shows that its Volmer–Heyrovsky dynamic process is faster. It is worth noting that the Ni_0.03_-MoS_2_-200 catalyst shows the best results in two key parameters (overpotential and Tafel slope): the overpotential is 208 mV and the Tafel slope is 122 mV/dec.

**Figure 9:**
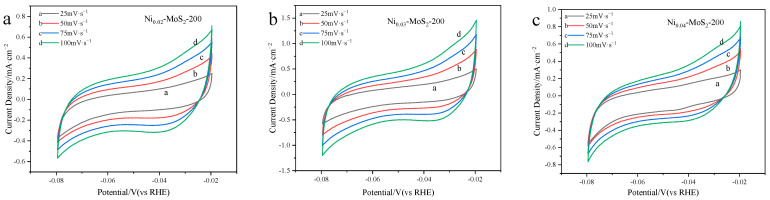
CV diagram of (**a**) Ni_0.02_-MoS_2_-200, (**b**) Ni_0.03_-MoS_2_-200, and (**c**) Ni_0.04_-MoS_2_-200 prepared with Ni doping ratios of 0.02, 0.03, and 0.04, respectively.

Figure 9 clearly shows the cyclic voltammetry (CV) of the Ni_0.02_-MoS_2_-200, Ni_0.03_-MoS_2_-200, and Ni_0.04_-MoS_2_-200 catalysts. It can be found that the current density on the cyclic voltammetry of the three materials shows a regular increase with the increase in the scanning speed. This shows that we can effectively adjust the microstructure of Ni-MoS_2_-200 and its performance in the water cracking reaction by adjusting the doping ratio of reactants. The experimental data show that when the molar ratio of raw material Ni to Mo is 3%, the properties of the material reach the best state.

**Figure 10:**
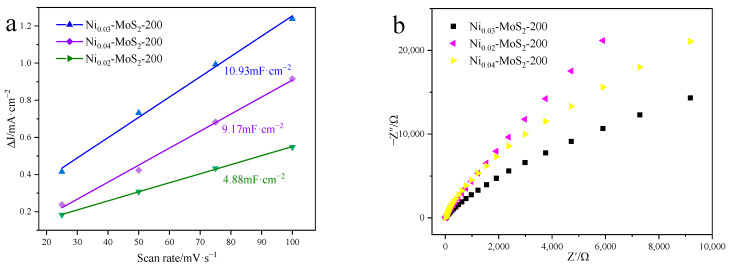
(**a**) C_dl_ diagram and (**b**) electrochemical impedance diagram of Ni-MoS_2_-200 prepared with different Ni doping ratios.

Figure 10a shows the electric double layer capacitance curves of Ni-MoS_2_-200 samples with different doping ratios (2%, 3%, and 4%) in detail. By fitting these curves and calculating their slopes, we find that their capacitance values are 4.88 mF/cm^2^, 10.93 mF/cm^2^, and 8.39 mF/cm^2^, respectively. It is particularly noteworthy that the capacitance C_dl_ of the Ni_0.03_-MoS_2_-200 sample reaches 10.93 mF/cm^2^, demonstrating that the Ni_0.03_-MoS_2_-200 material demonstrates excellent performance, improving reaction activity and electrocatalytic performance. Figure 10b further analyzes the impedance characteristics of these samples. Based the data in the figure, we find that the catalytic material Ni_0.03_-MoS_2_-200 has the smallest semicircle diameter, indicating that it is the lowest resistance in the charge transfer path, which makes it possible to more efficiently transfer electrons. On the whole, Ni_0.03_-MoS_2_-200 nano-materials have the best performance in improving activity and electrocatalytic performance.

A comprehensive analysis of all the materials studied, including Ni_0.03_-MoS_2_-190, Ni_0.03_-MoS_2_-200, Ni_0.03_-MoS_2_-210, Ni_0.02_-MoS_2_-200, and Ni_0.04_-MoS_2_-200, shows that the Ni_0.03_-MoS_2_-200 nano-materials have excellent electrocatalytic properties.

### 2.3. Characterization of Ni_0.03_-MoS_2_-200/GO and Study of HER Performance

#### 2.3.1. Characterization of Structure and Morphology of Ni_0.03_-MoS_2_-200/GO

**Figure 11:**
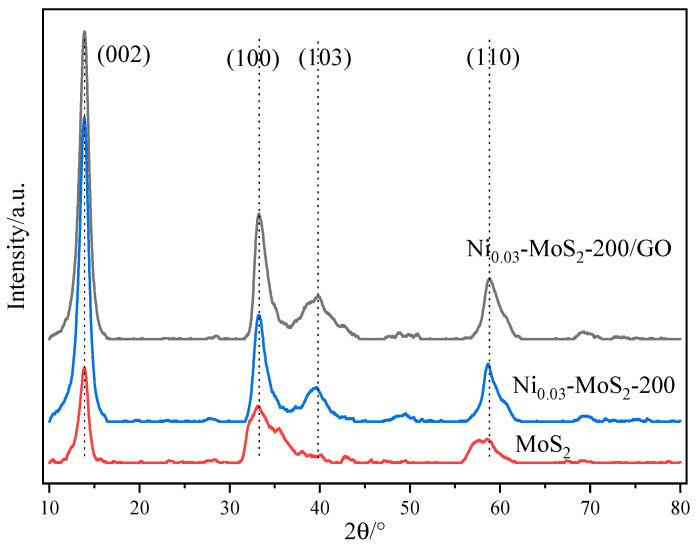
XRD patterns of MoS_2_, Ni_0.03_-MoS_2_-200, and Ni_0.03_-MoS_2_-200/GO.

Figure 11 shows the XRD patterns of MoS_2_, Ni_0.03_-MoS_2_-200, and Ni_0.03_-MoS_2_-200/GO. The outlines of these three curves are generally similar, but there are also slight differences. It shows that Ni doping and GO compounding have not seriously changed the crystal structure of MoS_2_. However, the peak intensity of the (103) and (110) crystal planes are quite different, with the minimum being MoS_2_, the middle being Ni_0.03_-MoS_2_-200, and the maximum being Ni_0.03_-MoS_2_-200/GO. In terms of crystallinity, MoS_2_ is the smallest, and Ni_0.03_-MoS_2_-200 and Ni_0.03_-MoS_2_-200/GO are almost the same. The exposure degree of the (103) and (110) crystal planes and the crystallization degree of the material may have an influence on the material performance. In addition, the diffraction peaks of Ni and GO are not clearly displayed in XRD, which is related to its low content.

**Figure 12:**
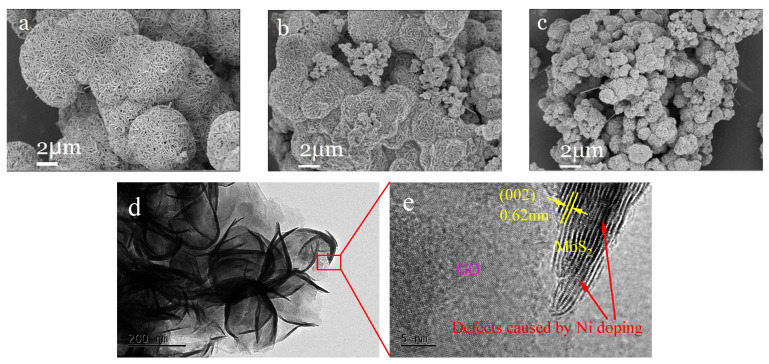

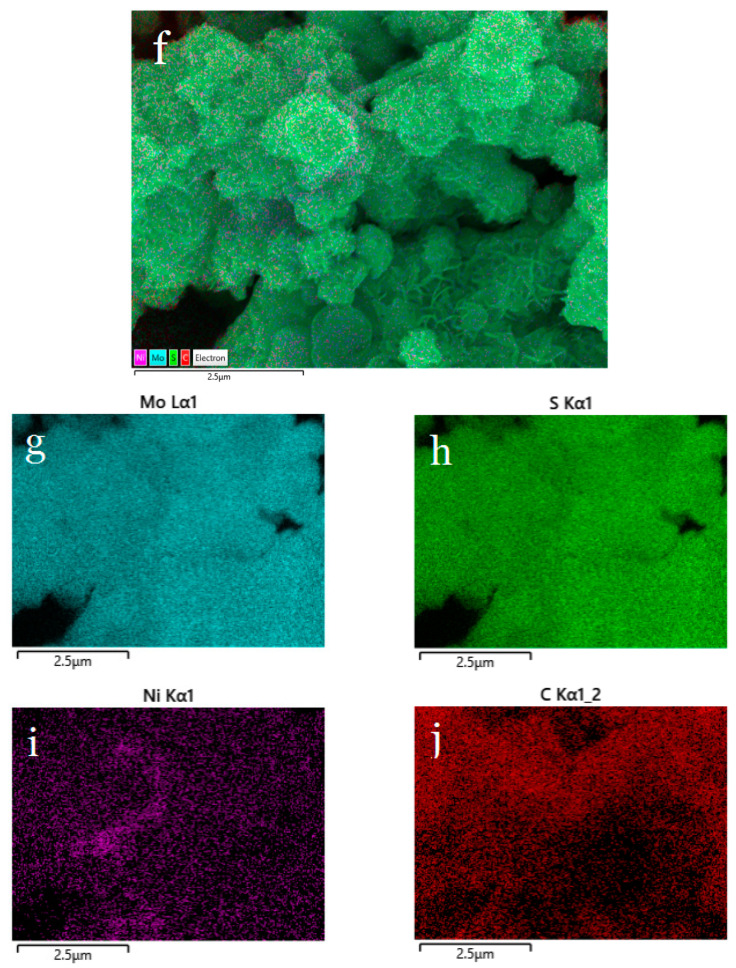

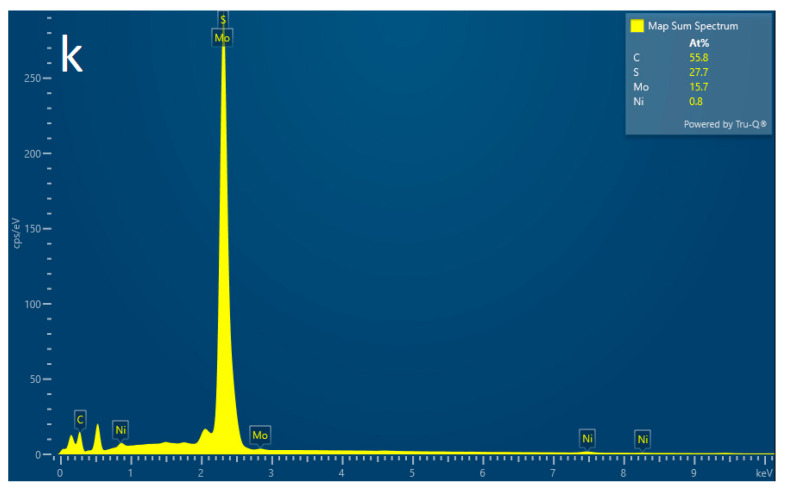
SEM images of (**a**) MoS_2_, (**b**) Ni_0.03_-MoS_2_-200, (**c**) Ni_0.03_-MoS_2_-200/GO, (**d**) TEM image and (**e**) HRTEM image and (**f**–**k**) EDS images of Ni_0.03_-MoS_2_-200/GO.

Figure 12 shows the scanning electron microscopy (Figure 12a–c) and transmission electron microscopy (Figure 12d,e) details of three different samples: MoS_2_, Ni_0.03_-MoS_2_-200, and Ni_0.03_-MoS_2_-200/GO. MoS_2_ is a regular nanosheet, and micron flower balls with a uniform size are formed by self-assembly. The overall morphology of Ni-doped MoS_2_(Ni_0.03_-MoS_2_-200) is similar to that of MoS_2_, but the regularity of nanosheets is weakened and the size of the micron flower balls is reduced, which leads to more defects, a larger specific surface area, and more active sites. Compared with Ni_0.03_-MoS_2_-200, Ni_0.03_-MoS_2_-200/GO formed after GO compounding possesses a smaller size and better dispersibility, which makes the specific surface area larger and provides more active sites. In addition, we have carried out energy spectrum scanning analysis on Ni_0.03_-MoS_2_-200/GO. As shown in Figure 12f–k, we can clearly see the existence of Mo, S, Ni, and C elements, which indicates that Ni_0.03_-MoS_2_-200/GO has been successfully prepared.

**Figure 13:**
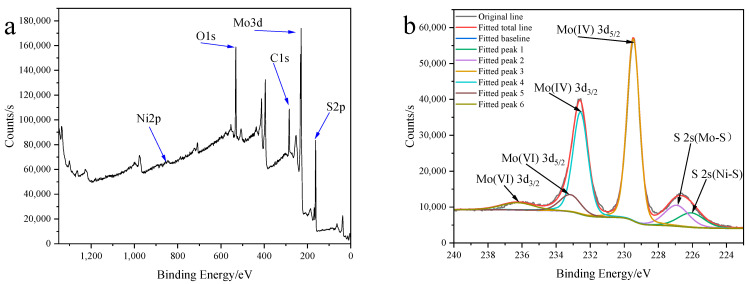

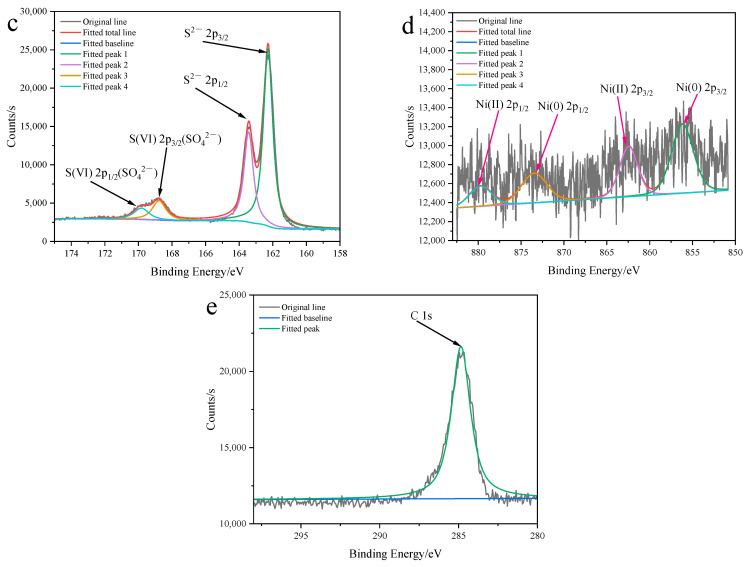
XPS spectrum of Ni_0.03_-MoS_2_-200/GO: (**a**) total, (**b**) Mo 3d and S 2s, (**c**) S 2p, (**d**) Ni 2p, (**e**) C 1s.

Figure 13 shows the XPS analysis results of the sample Ni_0.03_-MoS_2_-200/GO. It can be seen that the sample mainly contains Mo, S, C, O, and Ni (Figure 13a). As shown in Figure 13b, there are six characteristic peaks, of which the peak at 226.1 eV corresponds to S 2s of NiS, the peak at 226.9 eV corresponds to S 2s of MoS_2_, and the two peaks at 232.6 eV and 229.5 eV correspond to Mo(IV) 3d_3/2_ and Mo(IV) 3d_5/2_ of MoS_2,_ respectively. The peaks at 233.2 eV and 236.3eV correspond to Mo(VI) 3d_5/2_ and Mo(VI) 3d_3/2_ of MoO_3_, respectively, indicating that some Mo(IV) is oxidized to Mo(VI) [38,39,40]. Figure 13c shows the XPS diagram of S 2p, in which the peaks are 163.4 eV and 162 eV. The two characteristic peaks at 169.9 eV and 168.8 eV correspond to S(VI) 2p_1/2_ and S(VI) 2p_3/2_ of SO_4_^2−^, which indicates that some S elements are oxidized [38,41]. Figure 13d shows the XPS diagram of Ni 2p. There are two characteristic peaks at 856.2 eV and 873.5 eV, indicating the existence of Ni(0), and two characteristic peaks at 862.5 eV and 879.8 eV, indicating the existence of Ni(II). The result demonstrates that there are both NiS and elemental Ni in the sample [39,42]. Figure 13e shows the XPS diagram of C 1s, and the peak is located at 284.8 eV, which proves that the C = C bond is in graphene oxide [41,43].

#### 2.3.2. Performance Test of Electrocatalytic Hydrogen Evolution

**Figure 14:**
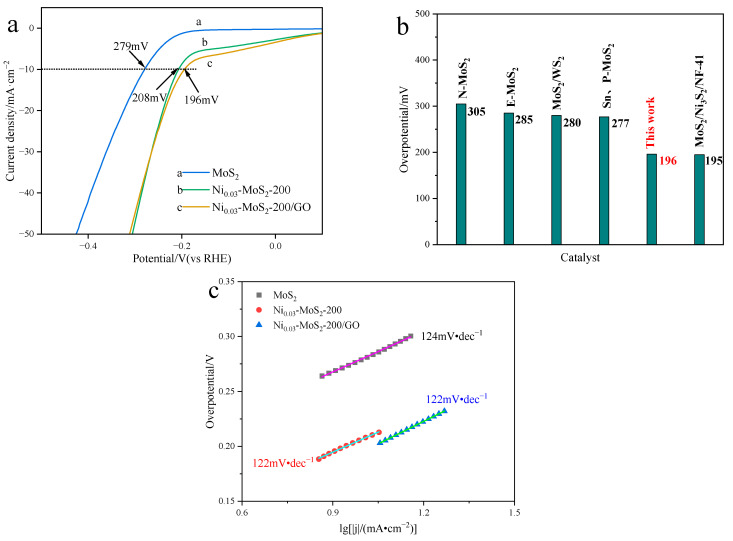
(**a**) LSV diagram (the black dotted line represents a current density of 10 mA·cm^−2^); (**b**) overpotential comparison diagram; (**c**) Tafel slope diagram of MoS_2_, Ni_0.03_-MoS_2_-200, and Ni_0.03_-MoS_2_-200/GO.

Figure 14a shows the LSV diagram of the materials MoS_2_, Ni_0.03_-MoS_2_-200, and Ni_0.03_-MoS_2_-200/GO. Compared with MoS_2_, Ni_0.03_-MoS_2_-200 presents a lower overpotential of 208 mV when the current density is 10 mA·cm^−2^, which is obviously lower than that of MoS_2_(279 mV), indicating that Ni doping effectively improves the efficiency of the electro-catalytic hydrolysis reaction (HER) of MoS_2_. When Ni_0.03_-MoS_2_-200 is further compounded with GO, the overpotential of the Ni_0.03_-MoS_2_-200/GO material formed by Ni_0.03_-MoS_2_-200 further decreases, reaching 196 mV, which is 12 mV lower than that of Ni_0.03_-MoS_2_-200, indicating that the compounding of GO with excellent conductivity improves the electron transmission rate of Ni_0.03_-MoS_2_-200 and further improves the hydrogen evolution performance. Compared with the studies (Figure 14b) [44,45,46,47,48] reported in recent years, its overpotential is relatively low. Figure 14c shows the Tafel slope diagrams of MoS_2_, Ni_0.03_-MoS_2_-200, and Ni_0.03_-MoS_2_-200/GO, respectively. Ni_0.03_-MoS_2_-200/GO and Ni_0.03_-MoS_2_-200 have lower Tafel slopes than MoS_2_. The Tafel slopes of Ni_0.03_-MoS_2_-200/GO and Ni_0.03_-MoS_2_-200 are equal, but the current density of Ni_0.03_-MoS_2_-200/GO is higher at the same overpotential, which shows that Ni_0.03_-MoS_2_-200/GO has better performance in the electrocatalytic hydrolysis reaction.

In this study, we have tested the cyclic voltammetry (CV) of three kinds of catalyst materials: MoS_2_, Ni_0.03_-MoS_2_-200, and Ni_0.03_-MoS_2_-200/GO. It is found that the current density of these materials on the CV curve shows a regular upward trend with the increase in scanning speed.

**Figure 15:**
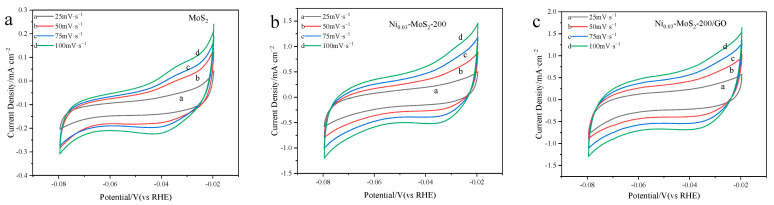
CV curves of (**a**) MoS_2_, (**b**) Ni_0.03_-MoS_2_-200, and (**c**) Ni_0.03_-MoS_2_-200/GO.

**Figure 16:**
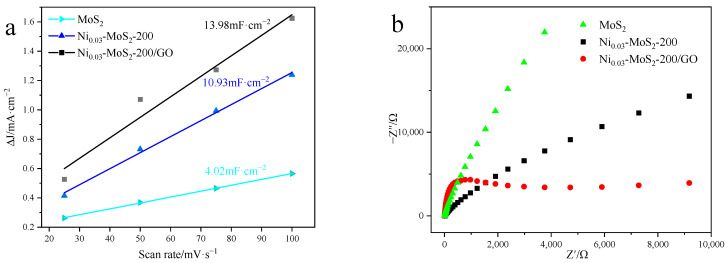
(**a**) C_dl_ diagram and (**b**) electrochemical impedance diagram of MoS_2_, Ni_0.03_-MoS_2_-200, and Ni_0.03_-MoS_2_-200/GO.

Based on the data in Figure 15, we obtained the C_dl_ of different catalysts, as shown in Figure 16a. The capacitance values of the three materials are 4.02 mF/cm^2^, 10.93 mF/cm^2^, and 13.98 mF/cm^2^, respectively. It is particularly noteworthy that the Ni_0.03_-MoS_2_-200/GO material has a high capacitance of 13.98 mF/cm^2^, which shows that Ni doping and GO compounding are beneficial to increasing the electrochemical active area and rich hydrogen evolution active sites of MoS_2_, so it has the best hydrogen evolution activity. Figure 16b reveals the impedance characteristics of three catalytic materials at different frequencies. Theoretically, the reduction in the semicircle diameter means that the resistance of charge transfer decreases in the electrocatalytic reaction, thus accelerating the reaction speed. The Ni_0.03_-MoS_2_-200/GO catalyst has the smallest semicircle radius, which shows that it encounters the least resistance in the process of charge mass transfer, so the electron transfer is more effective and the best catalytic activity is realized. To sum up, Ni doping and GO compounding together make MoS_2_ show the best performance.

**Figure 17:**
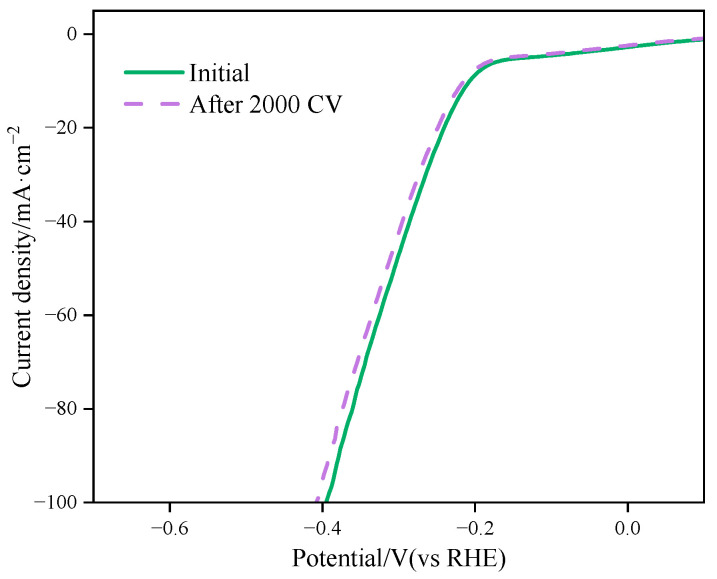
Stability of Ni_0.03_-MoS_2_-200/GO.

Another important criterion for evaluating catalysts is electrochemical stability. The Ni_0.03_-MoS_2_-200/GO complex with good hydrogen evolution performance was selected for the scanning cyclic voltammetry test, and then the changes in the linear voltammetry curves before and after scanning were compared. Figure 17 shows the LSV curves before and after scanning when Ni_0.03_-MoS_2_-200/GO is subjected to 2000 cycles of cyclic voltammetry in 0.5 mol/L H_2_SO_4_ at a scanning speed of 100 mV/s. It can be observed that the two curves are similar. The original overpotential is 196 mV with the current density at 10 mA/cm^2^. After 2000 cycles, the overpotential only increases to 198 mV. This result demonstrates that the prepared composite Ni_0.03_-MoS_2_-200/GO has good electrochemical stability.

## 3. Experimental Section

### 3.1. Reagents

Graphite powder, (NH_4_)_6_Mo_7_O_24_·4H_2_O, (NH_2_)_2_CS, NiCl_2_·6H_2_O, concentrated H_2_SO_4_, anhydrous ethanol, KMnO_4_, and 5 wt% Nafion are all analytical pure reagents and were purchased from Sinopharm Chemical Reagent Co., Ltd., (Shanghai, China).

### 3.2. Catalyst Synthesis

#### 3.2.1. Preparation of MoS_2_ and Ni-MoS_2_

In preparation, 3.1 g of (2.5 mmol) (NH_4_)_6_Mo_7_O_24_·4H_2_O and 4.7 g of (61.7 mmol) (NH_2_)_2_CS were weighed, dissolved in a beaker containing 100 mL deionized water, and ultrasonicated for 30 min. Then, 11.9 mg (0.050 mmol), 17.8 mg (0.075 mmol), and 23.8 mg (0.100 mmol) of NiCl_2_·6H_2_O were, respectively, added into another beaker, followed by adding 10 mL of deionized water and stirring for dissolution. Under magnetic stirring, the dissolved solution of NiCl_2_·6H_2_O was added into the first solution drop by drop, and ultrasound was continued for 30 min. Then, the mixed solution was poured into a 150 mL autoclave and heated at a preset reaction temperature (190 °C or 200 °C or 210 °C) for 24 h., followed by naturally cooling to room temperature, taking out, and washing with deionized water and ethanol. Finally, vacuum drying at 60 °C for 12 h occurred, and a black Ni_x_-MoS_2_ material was obtained. In the above hydrothermal reaction system, the molar ratios of Ni to Mo are 0.02:1, 0.03:1, and 0.04:1, respectively, and the obtained composite materials are named Ni_0.02_-MoS_2_, Ni_0.03_-MoS_2,_ and Ni_0.04_-MoS_2_, respectively. When the molar ratio of Ni to Mo is 0.03:1, the composite materials prepared at different temperatures (190 °C, 200 °C, or 210 °C) were named Ni_0.03_-MoS_2_-190, Ni_0.03_-MoS_2_-200, and Ni_0.03_-MoS_2_-210, respectively. As a contrast, a single MoS_2_ material was prepared under the same conditions without adding NiCl_2_·6H_2_O.

#### 3.2.2. Preparation of GO

At room temperature, 2 g of graphite and 1 g of sodium nitrate were accurately weighed and added into a 250 mL three-necked flask. The flask was cooled to near 0 °C in an ice bath. Then, we slowly added 50 mL of concentrated sulfuric acid under stirring and continued stirring for 30 min at a temperature not exceeding 5 °C. At 10 °C, we slowly added 0.3 g of potassium permanganate into the bottle and kept stirring for 30 min. Then, while keeping the temperature at 20 °C, 7 g of potassium permanganate was gradually added in three times. The cold bath was then removed and the reaction system was set to 35 °C by water bath, stirring for 2 h continuously. Then, 90 mL of water was slowly dropped into the brown suspension. We quickly raised the temperature, and when the system temperature rose to 90 °C, we continued to stir the suspension for 15 min. Then, 7 mL 30% hydrogen peroxide and 55 mL of distilled water (water temperature set at 45 °C) were added to the suspension. The suspension was filtered while it was hot, and the filter residue was washed three times with 3% dilute hydrochloric acid solution (volume 150 mL, temperature 45 °C). Next, the filter cake was dispersed in 600 mL of water. This was centrifuged at 4000 rpm for 20 min to separate gel-like graphene oxide. Finally, the filter cake was dried in a vacuum at 40 °C for 24 h, and GO was obtained.

#### 3.2.3. Preparation of Composite Material Ni_0.03_-MoS_2_-200/GO

First, 250 mg GO was dispersed in 100 mL deionized water and ultrasonicated for 2 h. Then, 3.1 g (2.5 mmol) (NH_4_)_6_Mo_7_O_24_·4H_2_O and 4.7 g (61.7 mmol) (NH_2_)_2_CS were mixed with the GO dispersion, and ultrasound was continued for 30 min. Then, 17.8 mg (0.075 mmol) of NiCl_2_·6H_2_O was dissolved in 10 mL of distilled water, and the solution was slowly dropped into the previous mixed solution under magnetic stirring, and ultrasonic treatment was continued for 30 min. Then, the mixed solution was transferred to a 150 mL reaction kettle, kept at a constant temperature of 200 °C for 24 h, and naturally cooled to room temperature. The product was centrifuged and washed with distilled water and ethanol at a speed of 10,000 rpm three times, each time for 8 min. Finally, the Ni_0.03_-MoS_2_/GO composite was prepared by vacuum drying at 60 °C for 24 h.

### 3.3. Catalyst Characterization

X-ray diffractometer (XRD-6100, Shimadzu Corporation, Kyoto, Japan) was used for phase analysis. Cu was used as the target, the tube voltage was 40 kV, and the tube current was 30 mA. The morphology was observed by using a Gemini SEM 500 scanning electron microscope (Carl Zeiss AG, Oberkochen, Germany), and the accelerating voltage was 2 kV. The energy spectrum was tested by the matching Oxford Aztec UltimMax 100 energy spectrometer (Oxford Instruments, Abington, UK). The high-resolution morphology was observed by the transmission electron microscopy (Tecnai G20, FEI Group, Hillsboro, OR, USA) and the accelerating voltage was 200 kV. X-ray photoelectron spectroscopy (XPS) was performed on a Thermo Scientific (Waltham, MA, USA) ESCALab 250Xi+ using 150 W monochromated Al Kα (1486.6 eV) radiation. A 500 µm X-ray spot was used for XPS analysis. The base pressure in the analysis chamber was about 3 × 10^−10^ mbar. Typically, the hydrocarbon C 1s line at 284.8 eV from adventitious carbon is used for energy referencing.

### 3.4. Electrocatalytic Hydrogen Evolution Test of Catalyst

In this study, the three-electrode system of the PGSTAT302N electrochemical workstation (Metrohm AG, Herrisau, Switzerland) was used to carry out the experimental study of electrocatalytic hydrogen evolution. During the experiment, a platinum sheet was used as the counter electrode, Ag-AgCl electrode (3.5 M KCl electrolyte solution) as the reference electrode, glassy carbon electrode coated with electrocatalyst as the working electrode, and the electrolyte was 0.5 mol/L H_2_SO_4_ solution. The experimental steps are as follows: First, remove the 4.0 mg sample and disperse it in a 1.0 mL mixed solution of water and ethanol (volume ratio is 7:3) containing 80 μL of 5% Nafion solution. After these substances were fully mixed, ultrasonic treatment was carried out for 1 h. Next, 5.0 μL of the uniform slurry containing electrocatalyst was sucked, smeared on a glassy carbon electrode with a diameter of 4.0 mm, and dried at 60 °C to form the final working electrode. In order to evaluate the electrocatalytic hydrogen evolution performance of the catalyst, we tested the linear sweep voltammetry, cyclic voltammetry, and electrochemical impedance of the catalyst. The electrochemical impedance spectroscopy (EIS) was measured at a frequency range from 10^5^ Hz to 0.01 Hz at a potential of −0.2 V vs. RHE. All the potentials were converted to the potential versus the reversible hydrogen electrode (RHE) according to the following equation: *E*_vs. RHE_ = *E* _vs. Ag/AgCl_ + 0.059 × pH + 0.2046, and this is compensated by iR.

## 4. Conclusions

The microstructure and electrocatalytic performance of the Ni-MoS_2_ nano-materials can be effectively improved by accurately adjusting the reaction temperature and the doping amount of Ni. When the molar ratio of Ni to Mo is 3% with the hydrothermal temperature of 200 °C, the prepared Ni_0.03_-MoS_2_-200 nanometer material shows excellent electrocatalytic performance: its η_10_ is as low as 208 mV, its Tafel slope is 122 mV/dec, and the electric double layer capacitance is 10.93 mF/cm^2^. By doping Ni, the electrocatalytic activity of MoS_2_ was improved, and its hydrogen evolution performance was significantly enhanced.

Ni_0.03_-MoS_2_-200/GO composites were successfully prepared by a hydrothermal method combined with GO composite technology. Due to the common impact of Ni doping and GO compounding, Ni_0.03_-MoS_2_-200/GO shows better catalytic activity than MoS_2_ and Ni_0.03_-MoS_2_-200, with lower overpotential (η_10_ = 196 mV) and a higher electric double layer capacitance of 13.98 mF/cm^2^. Through the strategy of GO compounding, the activity of MoS_2_ was improved and its catalytic performance was further enhanced

## Data Availability

Data are contained within the article.

## References

[1] Wickramaratne K.M.K., Ramezanipour F. (2024). Electrocatalytic properties of quasi-2D oxides LaSrMn_0.5_M_0.5_O_4_ (M = Co, Ni, Cu, and Zn) for hydrogen and oxygen evolution reactions. Molecules.

[2] Chen Q.Y., Tian S.Q., Liu X.N., An X.G., Zhang J.X., Xu L.H., Yao W.T., Kong Q.Q. (2022). Morphology-controlled synthesis of V_1.11_S_2_ for electrocatalytic hydrogen evolution reaction in acid media. Molecules.

[3] Meng C., Chen X.D., Gao Y.F., Zhao Q.Q., Kong D., Lin M.C., Chen X.M., Li Y.X., Zhou Y. (2020). Recent modification strategies of MoS_2_ for enhanced electrocatalytic hydrogen evolution. Molecules.

[4] Abdelghafar F., Xu X.M., Jiang S.P., Shao Z.P. (2022). Designing single-atom catalysts toward improved alkaline hydrogen evolution reaction. Mater. Rep.-Energy.

[5] Pehlivan L.B., Atak G., Niklasson G.A. (2021). Electrochromic solar water splitting using a cathodic WO_3_ electrocatalyst. Nano Energy.

[6] Jiang X.L., Jang H., Liu S.G. (2021). Heterostructure of Ru_2_P/WO_3_/NPC synergistically promotes H_2_O dissociation for improved hydrogen evolution. Angew. Chem. Int. Edit..

[7] Grzeszczuk M., Poks P. (2000). The HER performance of colloidal Pt nanoparticles incorporated in polyaniline. Electrochim. Acta.

[8] Liu X.T., Wang C.W. (2020). In situ wet etching of MoS_2_@dWO_3_ heterostructure as ultra-stable highly active electrocatalyst for hydrogen evolution reaction. Catalysts.

[9] Xu X.M., Pan Y.L., Zhong Y.J., Ge L., Jiang S.P., Shao Z.P. (2020). From scheelite BaMoO_4_ to perovskite BaMoO_3_: Enhanced electrocatalysis toward the hydrogen evolution in alkaline media. Compos. Part B-Eng..

[10] Chen Y., Yang K., Jiang B. (2017). Emerging two dimensional nanomaterials for electrochemical hydrogen evolution. J. Mater. Chem. A.

[11] Gupta U., Rao C. (2017). Hydrogen generation by water splitting using MoS_2_ and other transition metal dichalcogenides. Nano Energy.

[12] Xu X.M., Zhong Y.J., Wajrak M., Bhatelia T., Jiang S.P., Shao Z.P. (2024). Grain boundary engineering: An emerging pathway toward efficient electrocatalysis. InfoMat.

[13] Wang X., Yao X.L., Hou J.F. (2014). Non-isothermal crystallization of aqueous graphene oxide suspensions. J. Zhejiang Univ. (Eng. Sci.).

[14] Li Y.G., Wang H.L., Xie L.M. (2011). MoS_2_ nanoparticles grown on graphene: An advanced catalyst for the hydrogen evolution reaction. J. Am. Chem. Soc..

[15] Yin Y., Miao P., Zhang Y. (2017). Significantly increased raman enhancement on MoX_2_ (X = S, Se) monolayers upon phase transition. Adv. Funct. Mater..

[16] Tang L.H., Wang Y., Li Y.M. (2009). Preparation, structure, and electrochemical properties of reduced graphene sheet films. Adv. Funct. Mater..

[17] Lin W.J., Liao C.S., Jhang J.H. (2009). Graphene modified basal and edge plane pyrolytic graphite electrodes for electrocatalytic oxidation of hydrogen peroxide and β-nicotinamide adenine dinucleotide. Electrochem. Commun..

[18] Cao Y.Y., Wang L.F., Chen M.Y. (2021). W_2_N/WC composite nanofibers as an efficient electrocatalyst for photoelectrochemical hydrogen evolution. RSC Adv..

[19] Pham V.P., Yeom G.Y. (2016). Recent advances in doping of molybdenum disulfide: Industrial applications and future prospects. Adv. Mater..

[20] Xue J.Y., Li F.L., Zhao Z.Y. (2019). Roadmap and direction towards high performance MoS_2_ hydrogenevolution catalysts. Inorg. Chem..

[21] Zhao M.X., Yang M.Y., Huang W.J. (2021). Synergism on electronic structures and active edges of metallic vanadium disulfide nanosheets via Co doping for efficient hydrogen evolution reaction in seawater. ChemCatChem.

[22] Wang C., Wang S. (2019). Effect of Ni doping on electrocatalytic hydrogen evolution activity of MoS_2_. Int. J. Electrochem. Sci..

[23] Li M., Cai B., Tian R. (2021). Vanadium doped 1T MoS_2_ nanosheets for highly efficient electrocatalytic hydrogen evolution in both acidic and alkaline solutions. Chem. Eng. J..

[24] Yang H., Yuan M.W., Sun Z.M. (2020). In situ construction of a Mn^2+^-doped Ni_3_S_2_ electrode with highly enhanced urea oxidation reaction performance. ACS Sustain. Chem. Eng..

[25] Liu Q., Xie L.S., Liu Z.A. (2017). A Zn-doped Ni_3_S_2_ nanosheet array as a high-performance electrochemical water oxidation catalyst in alkaline solution. Chem. Commun..

[26] Bonde J., Moses P.G., Jaramillo T.F. (2008). Hydrogen evolution on nano-particulate transition metal sulfides. Faraday Discuss..

[27] Jaramillo T.F., Jorgensen K.P., Bonde J. (2007). Identification of active edge sites for electrochemical H_2_ evolution from MoS_2_ nanocatalysts. Science.

[28] Merki D., Vrubel H., Rovelli L. (2012). Fe, Co, and Ni ions promote the catalytic activity of amorphous molybdenum sulfide films for hydrogen evolution. Chem. Sci..

[29] Wang H., Tsai C., Kong D. (2015). Transition-metal doped edge sites in vertically aligned MoS_2_ catalysts for enhanced hydrogen evolution. Nano Res..

[30] Zhao M., Zhou G., Liu X., Shen X., Lv J., Hu C., Wang Y., Tan W., Sun S., Ma Y. (2021). One step hydrothermal synthesis of Ni-MoS_2_-RGO bifunctional electrocatalysts for HER and OER. Int. J. Electrochem. Sci..

[31] Chen L.X., Chen Z.W., Wang Y., Yang C.C., Jiang Q. (2018). Design of dual-modified MoS_2_ with nanoporous Ni and graphene as efficient catalysts for the hydrogen evolution reaction. ACS Catal..

[32] Lin C., Gao Z., Jin J. (2019). Boosting alkaline hydrogen evolution activity with Ni-doped MoS_2_/reduced graphene oxide hybrid aerogel. ChemSusChem.

[33] Yin X., Sun G., Song A., Wang L., Wang Y., Dong H., Shao G. (2017). A novel structure of Ni-(MoS_2_/GO) composite coatings deposited on Ni foam under supergravity field as efficient hydrogen evolution reaction catalysts in alkaline solution. Electrochim. Acta.

[34] Sun X., Dai J., Guo Y. (2014). Semimetallic molybdenum disulfide ultrathin nanosheets as an efficient electrocatalyst for hydrogen evolution. Nanoscale.

[35] Wang R., Yang Y., Sun Z. (2022). Ga doped Ni_3_S_2_ ultrathin nanosheet arrays supported on Ti_3_C_2_-MXene/Ni foam: An efficient and stable 3D electrocatalyst for oxygen evolution reaction. Int. J. Hydrogen Energy.

[36] Ahmad M., Ahmed E., Zhang Y. (2013). Preparation of highly efficient Al-doped ZnO photocatalyst by combustion synthesis. Curr. Appl. Phys..

[37] Huang Z., Luo W., Ma L. (2015). Dimeric [Mo_2_S_12_]^2−^ cluster: A molecular analogue of MoS_2_ edges for superior hydrogen-evolution electrocatalysis. Angew. Chem. Int. Edit..

[38] Wu L., Xu X., Zhao Y. (2017). Mn doped MoS_2_/reduced graphene oxide hybrid for enhanced hydrogen evolution. Appl. Surf. Sci..

[39] Wang D., Zhang X., Shen Y. (2016). Ni-doped MoS_2_ nanoparticles as highly active hydrogen evolution electrocatalysts. RSC Adv..

[40] Yin Y., Han J., Zhang Y. (2016). Contributions of phase, sulfur vacancies, and edges to the hydrogen evolution reaction catalytic activity of porous molybdenum disulfide nanosheets. J. Am. Chem. Soc..

[41] Ye J., Yu Z., Chen W. (2016). Ionic-liquid mediated synthesis of molybdenum disulfide/grapheme composites: An enhanced electrochemical hydrogen evolution catalyst. Int. J. Hydrogen Energy.

[42] Leyral G., Brillouet S., Rousseau J. (2017). Effect of the presence of ionic liquid during the NiMoS bulk preparation in the transformation of decanoic acid. Appl. Catal. A-Gen..

[43] Sahoo M., Ramaprabhu S. (2018). One-pot environment-friendly synthesis of boron doped graphene-SnO_2_ for anodic performance in Li ion battery. Carbon.

[44] Zheng X.J., Chen L.Y., Wang J.W., Zhu H.W., He W.Y. (2023). One-step synthesis and enhanced electrocatalytic hydrogen evolution performance of interlayer-expanded molybdenum disulfide. J. Mater. Eng..

[45] Zhou L., He W.Y., Chen L.Y., Zhu H.W., Chen L.J., Ling H., Zheng X.J. (2023). Preparation of Sn, P co-doped MoS_2_ nanoflowers and their electrocatalytic hydrogen evolution performance. Mater. Rep..

[46] Yang C.G., Huang R., Wang D.E., Tian Z.J. (2024). Electrocatalytic hydrogen evolution performance of nitrogen-doped molybdenum disulfide nanocatalysts. Chem. Ind. Eng. Prog..

[47] Wang D.Z., Yang L.Y.Y., Liu R.Q., Guo T., Fei H., Wu Z.Z. (2023). Preparation and electrocatalytic hydrogen evolution performance of spherical hollow MoS_2_/WS_2_ heterostructures. Trans. Nonferrous Metal. Soc..

[48] Jia F.H., Guo Y.C., Zou X.Y., Wei X.L., Bao W.W., Li Y. (2023). Highly efficient electrocatalytic hydrogen evolution behavior of MoS_2_/Ni_3_S_2_/NF in all-pH range. Fine Chem..

